# 538 Partial Thickness Pediatric Burn Injuries Treated with Autologous Skin Cell Suspension

**DOI:** 10.1093/jbcr/irac012.167

**Published:** 2022-03-23

**Authors:** Nicole M Kopari, Fabienne Gray, Herb A Phelan, Jeffrey E Carter

**Affiliations:** Children's Hospital New Orleans, New Orleans, Louisiana; Children's Hospital New Orleans, New Orleans, Louisiana; LSUHSC-New Orleans Department of Surgery, New Orleans, Louisiana; University Medical Center- New Orleans, New Orleans, Louisiana

## Abstract

**Introduction:**

Management of pediatric burn injuries resulting in optimal aesthetic remains a significant challenge in burn care. Wound care and acute surgical intervention coupled with reconstructive interventions is an essential component of burn care. Incorporation of new technologies in burn care has challenged historic paradigms. Our goal was to evaluate the use of autologous skin cell suspension (ASCS) for the treatment of partial-thickness pediatric burn injuries.

**Methods:**

A retrospective chart review from a single pediatric institution over a 10-month period was performed on patients undergoing treatment with ASCS. Patients with full-thickness injuries treated with autografting were excluded. Demographics and data collection included total burn surface area (TBSA), location of burn, mechanism of burn, time to ASCS application, time to >90% re-epithelization, hospital length of stay, ASCS failure requiring repeat operation, and reconstructive procedures or laser interventions.

**Results:**

26 pediatric patients ≤13 years of age charts were reviewed. 14 patients received ASCS and met inclusion criteria. 8 faces were included in our study along with 11 upper extremity burns, 5 lower extremity burns, and 8 torso burns or some combination of the above. The most common etiology was scald injury from hot water followed by noodle soup burns and grease burns. Other etiologies included road rash, flame burn, and a steam burn. ASCS was applied 2 days (range 1-4) after injuries and patients only required 1 operation. The average length of hospital stay was 4 days (range 1-10) and the average TBSA was 10% (range 4-17). The average time to >90% re-epithelization was 7 days with one outlier with healing at day 24. This is the only patient in the ASCS group that required laser interventions. No patients required repeat procedures, subsequent autografting, or reconstructive procedures.

**Conclusions:**

Pediatric patients with partial-thickness burns benefitted from the ASCS by having limited donor sites, short hospitalizations compared to %TBSA, improved time to >90% re-epithelization, and no repeat surgical interventions. The fast-healing time and good cosmetic outcome decreases the need for compression garments and subsequent laser interventions. Key factors include patient selection and appropriate wound preparation.

